# The Antibacterial Activities and Characterizations of Biosynthesized Zinc Oxide Nanoparticles, and Their Coated with Alginate Derived from *Fucus vesiculosus*

**DOI:** 10.3390/polym15102335

**Published:** 2023-05-17

**Authors:** Ragaa A. Hamouda, Asrar A. Alharbi, Majdah M. Al-Tuwaijri, Rabab R. Makharita

**Affiliations:** 1 Department of Biology, College of Science and Arts at Khulis, University of Jeddah, Jeddah 21959, Saudi Arabia or soso1431h@gmail.com (A.A.A.); rabab_makharita@science.suez.edu.eg (R.R.M.); 2 Genetic Engineering and Biotechnology Research Institute (GEBRI), University of Sadat City, Sadat City 32897, Egypt; 3 Department of Biology, Faculty of Applied Science, Umm-Al-Qura University, Makkah Al-Mukarramah 21955, Saudi Arabia; mmtuwaijri@uqu.edu.sa; 4 Botany and Microbiology Department, Faculty of Science, Suez Canal University, Ismailia 41522, Egypt

**Keywords:** *Fucus vesiculosus*, ZnO-NPs, alginate, characterizations, antibacterial

## Abstract

Zinc oxide nanoparticles have many advantages for nano-biotechnologists due to their intense biomedical applications. ZnO-NPs are used as antibacterial agents, which influence bacterial cells through the rupture of the cell membrane and the generation of reactive free radicals. Alginate is a polysaccharide of natural origin due to its excellent properties that are used in various biomedical applications. Brown algae are good sources of alginate and are used as a reducing agent in the synthesis of nanoparticles. This study aims to synthesize ZnO-NPs by using brown alga *Fucus vesiculosus* (Fu/ZnO-NPs) and also to extract alginate from the same alga, which is used in coating the ZnO-NPs (Fu/ZnO-Alg-NCMs). The characterizations of Fu/ZnO-NPs and Fu/ZnO-Alg-NCMs were determined by FTIR, TEM, XRD, and zeta potential. The antibacterial activities were applied against multidrug resistance bacteria of both gram-positive and negative. The results obtained in FT-TR showed there are some shifts in the peak positions of Fu/ZnO-NPs and Fu/ZnO-Alg-NCMs. The peak at 1655 cm^−1^, which assigned amide I-III, is present in both Fu/ZnO-NPs and Fu-Alg-ZnO-NCMs; this band is responsible for bio-reductions and stabilization of both nanoparticles. The TEM images proved the Fu/ZnO-NPs have rod shapes with sizes ranging from 12.68 to 17.66 and are aggregated, but Fu/ZnO/Alg-NCMs are spherical in shape with sizes ranging from 12.13 to 19.77. XRD-cleared Fu/ZnO-NPs have nine sharp peaks that are considered good crystalline, but Fu/ZnO-Alg-NCMs have four broad and sharp peaks that are considered semi-crystalline. Both Fu/ZnO-NPs and Fu/ZnO-Alg-NCMs have negative charges (−1.74 and −3.56, respectively). Fu/ZnO-NPs have more antibacterial activities than Fu/ZnO/Alg-NCMs in all tested multidrug-resistant bacterial strains. Fu/ZnO/Alg-NCMs had no effect on *Acinetobacter* KY856930, *Staphylococcus epidermidis*, and *Enterobacter aerogenes*, whereas there was an apparent effect of ZnO-NPs against the same strains.

## 1. Introduction

Bacterial infections are accredited as a severe health problem on a global scale. The need for more effective antibacterial agents is growing as a result of rising pathogenic strain outbreaks, antibiotic resistance, and new bacterial mutations. Since ancient times, zinc oxide has been used for its antimicrobial properties [1]. Nanomaterials with antibacterial action, such as metal nanoparticles and metal oxide nanoparticles, are currently considered a new line of defense against bacterial diseases. These materials have a unique antibacterial mechanism due to the nanoscale surface effect and small size effect, which primarily involves three points of view: release of metal ions or reactive oxygen species (ROS) to damage bacterial proteins and DNA; nanoparticles aggregate and destroy bacterial cell membranes [2]. Over the past 20 years, ZnO-NPs have become one of the most popular metal oxide nanoparticles in biological applications because of their excellent biocompatibility and low toxicity. ZnO-NPs can be used as an antibacterial material due to their outstanding properties, such as a large specific surface area and robust activity to inhibit a range of pathogenic pathogens [3]. ZnO-NPs are good for antibacterial use due to their outstanding biocompatibility, photochemical stability, and other features [4].

The ZnO-NP has antifungal potency against *Penicillium expansum, Botrytis cinerea* [5], *Aspergillus*, *Penicillium* [6], and *Candida albicans* [7]. The ZnO-NPs deform fungus hyphae, which prevent *Botrytis cinerea* from proliferating, suppress the development of conidiophores and conidia and kill off fungal hyphae of *Penicillium expansum* [5].

ZnO-NPs have traditionally been created using a variety of chemical techniques [8], but the processes produce a lot of dangerous by-products. Green nanotechnology, which includes a safe, non-toxic, and environmentally friendly method of nanoparticle synthesis, is required. Due to its ease, environmental friendliness, and robust antibacterial activity, plant-mediated biological production of nanoparticles is currently gaining popularity [9].

Marine macroalgae are the most pleasant and promising algae group to supply innovative biochemically active compounds for this reason of their broad spectrum of biological actions like antiviral [10], antimicrobial [11], antifungal [12], anticoagulant [13], anti-allergic [14], antifouling, antioxidant, and medical activities [15].

Using algal extracts to fabricate ZnO-NPs can have powerful anti-pathogenic properties on several deadly infectious illnesses [16]. The ZnO-NPs derived by brown alga *Cystoseira crinita* had good antimicrobial activity against gram-positive and negative bacteria and fungi such as *Candida albicans* and *Aspergillus niger* [17]. The ZnO-NPs biogenic by brown marine alga *Sargassum muticum* are expected in some applications such as the bio-medicals area, cosmetics, and pharmaceuticals [18]. The ZnO-NPs biosynthesized using marine alga *Gracilaria edulis* possessed anticancer activities versus PC3 cell lines [19].

Alginates are generated industrially from marine macroalgae, especially brown algae. The need for alginate generation has grown over time and is anticipated to grow further going forward [20]. Cell walls of marine brown algae contain polysaccharide anion (alginate), a natural, widely-used, biodegradable, and mucoadhesive polymer without toxicity in administration [21]. Alginates are thought to be materials that are non-immunogenic, biocompatible, and non-toxic [22]. Pharmaceuticals with alginate positions are used to treat the symptoms of oesophagitis and heartburn [23]. To enhance the qualities of alginate fibers, various materials were combined with alginate, such as inorganic substances, ceramics, and chitosan [24]. The combination of ZnO-NPs enhanced the rheological assets of the alginate. This is endorsed by the electrostatic interactions and intermolecular hydrogen bonding between zinc oxide nanoparticles and the alginate polysaccharide [25]. The ZnO-NP-alginate beads were extremely hopeful for various water treatment appliances, particularly for point-of-use in the perspectives of reusability, ZnO antibacterial capability, immobilizing NPs, and using the high surface area of NPs, with a minimal liberation of zinc ions [26]. The use of ZnO NPs and alginate offers an effective substitute for currently available chronic wound healing therapies [27]. Motelica et al. [28] reported the use of alginate/ZnO/citronella essential oil films as antimicrobial packaging for cheese to increase its shelf life.

The study aims to extract alginate from brown alga *F. vesiculosus*, bio-synthesized Zinc oxide nanoparticles using brown alga *F. vesiculosus* (Fu-ZnO-NPs), and coated Zinc oxide nanoparticles by alginate to form alginate/zinc oxide nano-composites (Fu-Alg-ZnO-NCMs). We will also assess the antibacterial activity of both Fu-ZnO-NPs and Fu-Alg-ZnO-NCMs.

## 2. Material and Methods

### 2.1. Alga

*Fucus vesiculosus* brown alga was gathered in April 2020 from the beach in Jeddah, Saud Arabia, and was recognized according to Taylor [29]. Alga was cleaned, dried in an oven at 60 °C for two days, ground, and sieved; the size of alga particles was 1–1.2 mm.

### 2.2. Green Synthesis of Zinc Oxide Nanoparticles

Under continual stirring, 80 mL of D water was supplied with zinc acetate dehydrate (0.02 M). Twenty mL of *F. vesiculosus* alga extract (1 gm in 100 D water boiling for one hour, and filtrate) was added to the zinc acetate mixture drop-wise, with stirring, and then NaOH (2.0 M) was added until the pH was 12, the mixture was then left for one hour. The precipitate was filtered, and the residue was washed two times with D water and one time with ethanol; the washing was conveyed by centrifugation. The precipitate (Fu-ZnO-NPs) was dried in the oven at 60 °C [30].

### 2.3. Alginate Extraction and Characterizations

The alginate extracted from *F. vesiculosus* was according to the methods of Hambali et al. [31]. The *F. vesiculosus* alginate was characterized according to previous research by Hamouda et al. [32].

### 2.4. Synthesis of Fu-Alg-ZnO-NCMs

*F. vesiculosus* sodium alginate in the amount of 1 gm was dissolved in 50 mL of D water at 40 °C, 0.037 gm of Fu-ZnO-NPs was added, and the mixture was agitated for 2 h before centrifuging, and drying the precipitate at 60 °C.

### 2.5. Characterizations of Fu/ZnO-NPs, and Fu/Alg-ZnO-NCMs

#### 2.5.1. Fourier Transform Infrared (FT-IR)

The Fu/ZnO-NPs and Fu/Alg-ZnO-NCMs active groups were assessed using a Fourier transform infrared spectrometer (FT-IR) (JASCO Europe S.r.l., Cremella, Italy), FT-IR 5300 spectrophotometer; the FT-IR spectrum ranged between 4000 and 400 cm^−1^.

#### 2.5.2. Transmission Electron Microscopy (TEM)

Particle size and shape of Fu-ZnO-NPs and Fu-Alg-ZnO-NCMs were characterized via transmission electron microscopy (JEOL JSM-6510/v, Tokyo, Japan), (TEM).

#### 2.5.3. X-ray Powder Diffraction (XRD)

The crystalline of nanoparticles was determined by an X-ray diffractometer (PAN Analytical X-Pert PRO, spectris plc, Almelo, The Netherlands); the Fu-ZnO-NPs and Fu-Alg-ZnO-NCMs size was determined using Scherrer’s equation.

(1) $$Crystal\;Size\;L=\frac{\lambda k}{\beta (\cos\theta )}$$

where λ = 0.1540 nm, k is the constant factor of 0.91, θ = diffraction angle in radians, and β = full width at half maximum (FWHM).

#### 2.5.4. Zeta Potential Analysis

Zeta Potential Analysis provided details of the stabilization of Fu-ZnO-NPs and Fu-Alg-ZnO-NCMs and was measured by Malvern Zeta size Nano-Zs90 (Malvern, Westborough, PA, USA).

#### 2.5.5. Energy-Dispersive Spectroscopy (EDS)

The surface morphology and elemental composition of both Fu-ZnO-NPs and Fu-Alg-ZnO-NCMs were determined by using a field emission scanning electron microscope equipped with energy-dispersive spectroscopy (EDS) (JEOL JSM-6510/v, Tokyo, Japan). 

### 2.6. Antibacterial Activities

The antibacterial activity of Fu/ZnO-NPs and Fu/Alg-ZnO-NCMs was displayed against multidrug-resistant bacteria (MDR) [33,34]. Gram-negative *Klebsiella pneumoniae* KY856924, *Acinetobacter* KY856930, *E. coli* KY856932, *E. coli* KY856933, *Enterobacter* KY856934, and *Enterobacter aerogenes* which have been isolated from different medical samples and identified as multidrug-resistant in a previous study [33,34], and Gram-positive control strains (*Staphylococcus aureus* ATCC 6538, *Staphylococcus aureus* ATCC 25923, *Staphylococcus epidermidis* ATCC 12228, *Streptococcus mutans* ATCC 25175). All the bacterial strains were cultured in nutrient broth at 37 °C for 24 h. A sterile cotton swab was used to spread the bacterial strains on Mueller-Hinton agar (MHA). Wells were made on the agar plates and filled with 100 µL of each Fu/ZnO-NPs and Fu/Alg-ZnO-NCMs (1 mg/mL). After 24 h of incubation at 37 °C, the clear zones were measured.

### 2.7. Statistical Analysis

Data of the effect of Fu/ZnO-NPs and Fu-Alg-ZnO-NCMs on different bacterial pathogens are presented as the mean standard error (SE) and were subjected to statistical analysis using one-way analysis of variance (ANOVA). The post hoc differences between means were tested by a Duncan multiple comparison test. Differences at *p* < 0.05 were considered significant. 

## 3. Results and Discussion

### 3.1. Fourier-Transform Infrared Spectroscopy (FTIR) Analysis of Fu/ZnO-NPs and Fu-Alg-ZnO-NCMs Bio-Fabricated via F. vesiculosus

**Figure 1:**
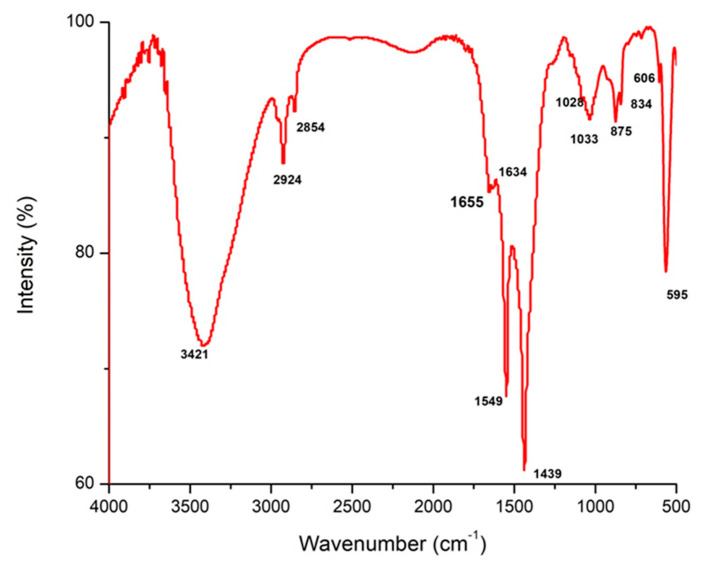
FT-IR analysis of Fu/ZnO-NPs bio-synthesized via brown alga *F. vesiculosus*.

**Figure 2:**
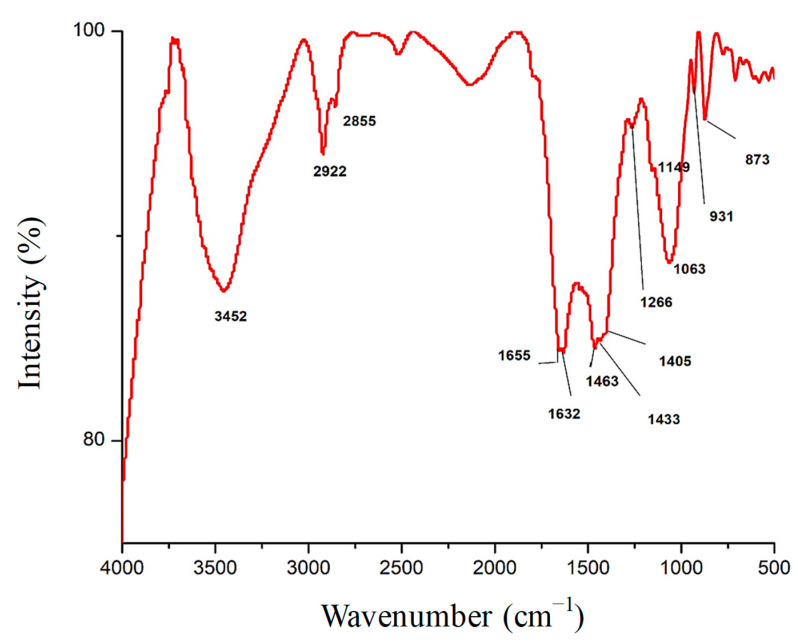
FT-IR analysis of Fu-Alg-ZnO-NCMs bio-synthesized via brown alga *F. vesiculosus*.

The results in Figure 1 and Table 1 demonstrate FT-IR spectroscopy analysis of Fu/ZnO-NPs bio-fabricated via *F. vesiculosus*. Findings appear to have 14 peaks in wavenumber regions of 3421, 2924, 2854, 1655, 1634, 1549.1439.1082, 1033, 875, 843, 606, 565, and 433 cm^−1^. Table 1 indicates FT-IR analysis of Fu-Alg-ZnO-NCMs, bio-synthesized via brown alga *F. vesiculosus*. The outcomes reveal that there are 18 bands with wavenumbers 3452, 2922, 2855, 1655, 1632, 1463, 1433, 1405, 1266, 1149, 1063, 931, 873, 710, 582, 532, and 472 cm^−1^ (Figure 2). A band at 1655 cm^−1^ is present in both Fu/ZnO-NPs and Fu-Alg-ZnO-NCMs; this band is responsible for bio-reductions and stabilization of Fu/ZnO-NPs. The results also demonstrated that some peaks were shifted to higher-frequency positions, and others shifted to lower-frequency positions; those variations related to the change of composition of Fu/ZnO-NPs and Fu/Alg-ZnO-NCMs.

### 3.2. XRD of Fu/ZnO-NPs, and Fu-Alg-ZnO-NCMs 

**Figure 3:**
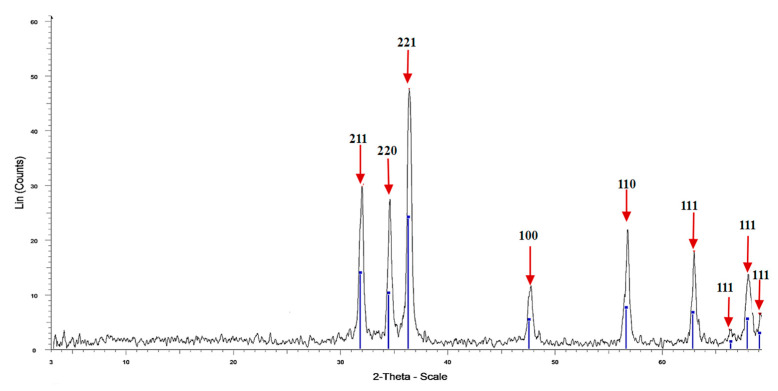
X-ray diffraction analysis of Fu/ZnO-NPs bio-fabricated via *F. vesiculosus*.

The results in Figure 3 and Table 2 show the X-ray diffraction analysis of Fu/ZnO-NPs bio-fabricated via *F. vesiculosus*. The results investigate nine sharp peaks at 2 theta 31.97, 34.57, 36.38, 47.74, 56.81, 63.33, 68.07, and 69.17° correspond to lattice plane (hkl) 211, 220, 220, 221, 110, 110, 111, 111, 111, and 111. The major crystalline peak was obtained at 2 Theta 36.388°, and the minor crystalline peak was obtained at 2 Theta 69.17°. All peaks of Fu/ZnO-NPs are sharp peaks, which denote the Fu/ZnO-NPs are crystalline and also denote the purity of ZnO-nanoparticles. The crystal size has been intended from XRD analysis by the Debye–Scherrer equation, which in this work was equal to 29.9 nm with 100% intensity. The peaks of Zn-GNPs obtained by XRD are the sharp and crystalline structure of the nanoparticles [56]. The XRD diffraction patterns of *Cystoseira crinite-* ZnO-NPs were assigned at 2θ values of 31.78, 34.42, 36.26, 47.6, 56.6, 62. 8, and 68° [46]. No peak at 20- 30 2θ could indicate the ZnO-NPs are pure [46]. The XRD of *Arthrospira platensis*-ZnO-NPs exhibited seven recognized peaks at 2 theta degree 31.7°, 34.5°, 36.1°, 47.4°, 56.3°, 63.1°, and 67.9° [57]. The XRD of *Chlorella*-ZnONPs exhibited eight recognized peaks at 2-Theta 31.8, 34.47, 36.29, 47.56, 56.59, 63.01, 67.94, and 69.07° [58].

**Figure 4:**
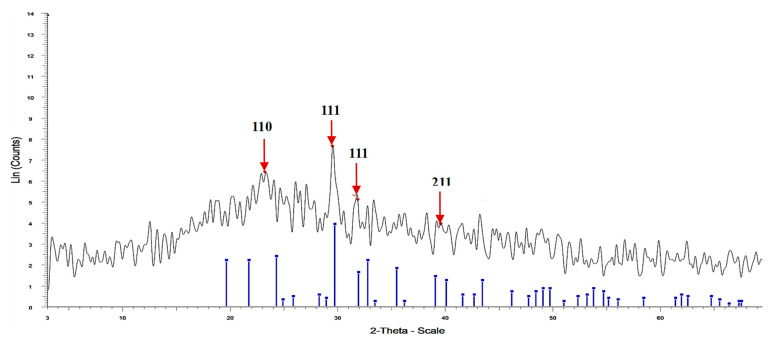
XRD analysis of Fu/ZnO/Alg-NCMs bio-fabricated by *F. vesiculosus*.

Figure 4 and Table 3 show the XRD patterns of Fu/Alg-ZnO-NCMs bio-synthesized via *F. vesiculosus*. The peaks were shown at four positions: 23.159, 29.457, 31.844, and 39.564, and reflect the (hkl) miller index at 110, 111, 111, and 211, respectively. The broad and sharp peaks denote Fu/ZnO/Alg-NCMs bio-synthesized via *F. vesiculosus* are semi-crystalline. The X-ray diffraction (XRD) results indicate that sodium alginate-ZnO nano-composites display a semi-crystalline nature [59]. The crystal size of Fu/ZnO-Alg-NCMs has been intended from XRD analysis by the Debye–Scherrer equation, which in this work was equal to 21.7 nm with 100% intensity. The average crystallite size for ZnO-NPs synthesized by leaf extracts of *Eucalyptus globulus* was 27 nm [60].

### 3.3. EDS Analysis of Fu/ZnO-NPs and Fu-Alg-ZnO-NCMs 

**Figure 5:**
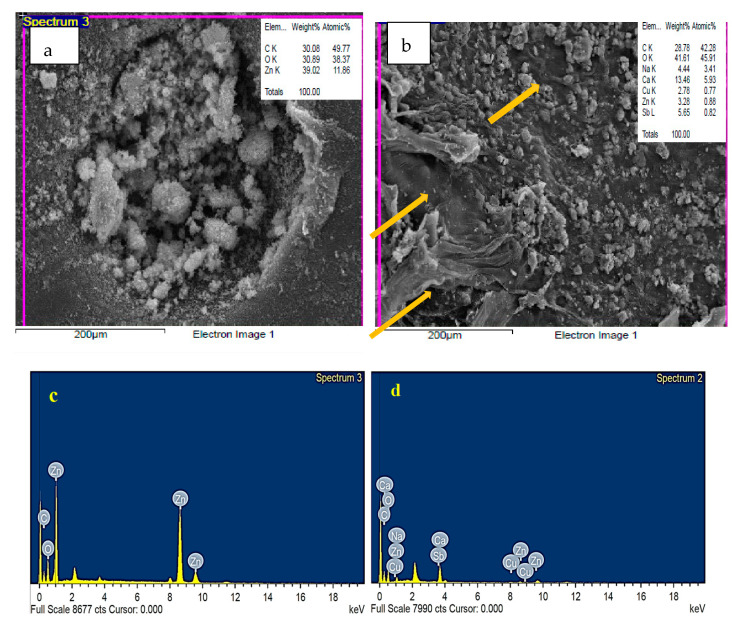
Scanning Electron Microscopic image (**a**,**b**), Energy dispersive X-ray spectrophotometry analysis (**c**,**d**) of bio-fabricated Fu/ZnO-Alg-NCMs and Fu/ZnO-NPs by *F. vesiculosus*.

The surface topography of bio-fabricated Fu/ZnO-Alg-NCMs and Fu/ZnO-NPs by *F. vesiculosus* was observed by Scanning Electron Microscopic (Figure 5). The results demonstrate Fu/ZnO-NPs (Figure 5a) have rough surfaces, and the particles are agglomerated, but in the case of Fu/ZnO-Alg-NCMs, Figure 5b shows the particles are irregularly spherical and impeded in alginate fibers as pointed by arrows. The EDS analysis in Figure 5c demonstrates the presence of elements Zn, C, and O by weight % of 30.08, 30.89, and 39.02, which confirmed the presence of Zn and oxygen. EdS analysis in Figure 5d demonstrates that the Zn is present in a low weight percentage (3.28%), and oxygen and carbon are present in high weight percentages (41.61 and 28.78, respectively), which confirmed the presence of alginate, and also the presence of Na and Ca proved the presences of alginate. Keshavarz et al. [61] reported the presence of a sodium peak in EdS analysis, proving the presence of alginate. The EDS spectra peak in both Fu/ZnO-NPs and Fu/ZnO-Alg-NCMs; the O peak appears at 0.5 KeV. Meanwhile, Zn peaks appear at 1 KeV, 8.6 KeV, and 9.5 KeV; these results are in agreement with [62]. Results of EDS spectra approve and verify the XRD result, which reveals the ability of *F. vesiculosus* marine macroalga to synthesize ZnO-NPs. 

### 3.4. TEM Image

**Figure 6:**
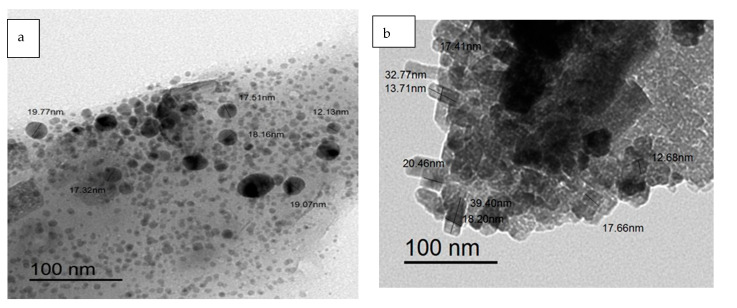
Transmission electron microscopic (TEM) image of bio-fabricated Fu/ZnO-Alg-NCMs (**a**) and Fu/ZnO-NPs (**b**).

The distribution and shape of Fu/ZnO-NPs and Fu/ZnO-Alg-NCMs that were bio-fabricated by brown alga *F. vesiculosus* were estimated by TEM, as shown in Figure 6. The results showed that Fu/ZnO/Alg-NCMs are spherical, and alginate is a core-shell around the zinc nanoparticles. The size of Fu/ZnO/Alg-NCMs ranged from 12.13 to 19.77 nm Figure 6a. Figure 6b shows the TEM image of Fu/ZnO-NPs, which displays that Fu/ZnO-NPs are rod in shape and size ranging from 12.68 to 17.66 related to width. The Bio-Zn-NPs via *Catharanthus roseus* are poly-dispersed with a particle size range of 10–20 nm [63]. The Bio-Zn-NPs were fabricated via edible mushrooms, are poly-dispersed with a particle size range of 12–17 nm, and have mostly spherical shapes [64]. TEM results confirmed biosynthesis of ZnO-NPs by *Anacardium occidentale* was hexagonal with a particle size of 33 nm [65]. According to the TEM image, ZnO-NPs bio-fabricated by pumpkin seeds extract are spherical in shape, with 48–50 nm [66]. ZnO-NPs bio-fabricated via *Ulva fasciata* were spherical and crystalline with a size range of 3–33 nm [67].

**Figure 7:**
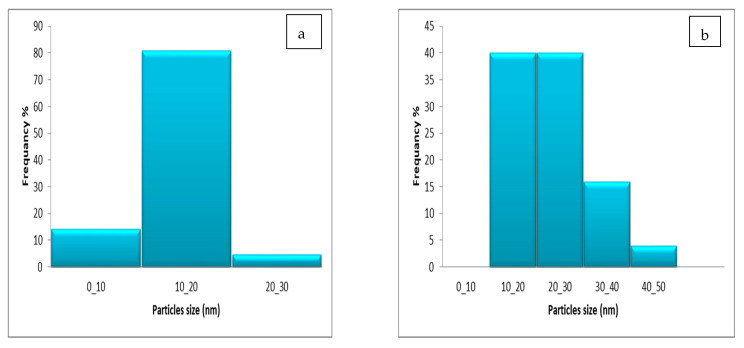
Particle size distribution of bio-fabricated Fu/ZnO-Alg-NCMs (**a**) and Fu/ZnO-NPs (**b**).

The most frequent Fu/ZnO-Alg-NCMs were 80% at a range size of 10–20 nm. Meanwhile, the most frequent Fu/ZnO-NPs were at 10–20 and 20–30 nm. These results denote that the alginates when capped with ZnO-NPs, changed from rod to spherical in shape and lowered in size (Figure 7a,b). Trandafilovic et al. [68] demonstrated several spherical particles evenly distributed throughout the alginate matrix. The sodium alginate contains evenly dispersed nano-ZnO [69]. The shapes of ZnO/WG (wheat gliadin) nanospheres were nearly spherical [70].

### 3.5. Zeta Potential Analysis

**Figure 8:**
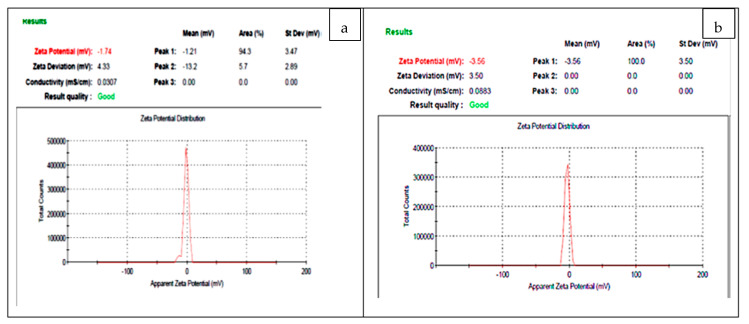
Zeta potential analysis of bio-fabricated Fu/ZnO-NPs (**a**) and Fu/ZnO/Alg-NCMs (**b**) bio-fabricated via brown alga *F. vesiculosus*.

Results obtained in Figure 8 demonstrate the zeta potential analysis of bio-fabricated Fu/ZnO-NPs and Fu/ZnO/Alg-NCMs bio-fabricated via brown alga *F. vesiculosus* have negative values (−1.74 and −3.56, respectively). If the value of the zeta potential ranges between 0 and ±5, it is an indicator for rapid coagulation ±10 to ±30, incipient instability ±30 to ±40, moderate stability, ±40 to ±60 good stability, and > ±61 mV excellent stability [71]. So, these results represent the Fu/ZnO-NPs and Fu/ZnO/Alg-NCMs bio-fabricated via brown alga *F. vesiculosus* are rapid coagulation, as in the TEM image. According to Haider and Mehdi 2014 [72], the zeta potential’s negative charge value demonstrates the effectiveness of capping agents in stabilizing AgNPs by indicating more negative charges that keep all the particles apart from one another. Negative values demonstrate the attraction between the particles, leading to a more stable AgNP structure that prevents agglomeration in aqueous solutions [73].

### 3.6. Antibacterial Activities of Fu/ZnO-NPs and Fu/ZnO-Alg-NCMs Bio-Fabricated via Brown Alga F. vesiculosus

**Figure 9:**
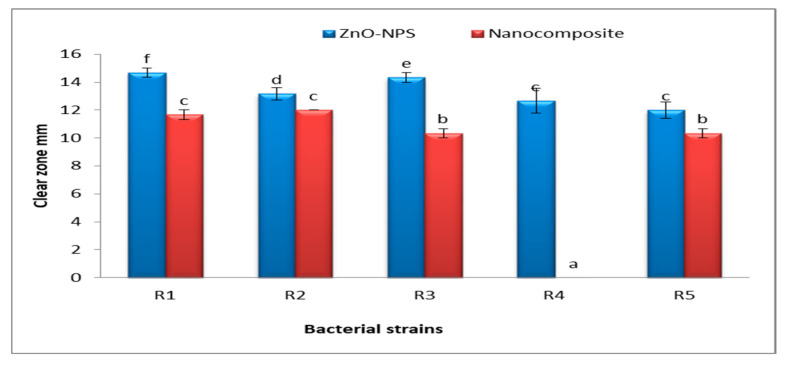
Antibacterial of Fu/ZnO-NPS and Fucus/ZnO/Alg-NCMs bio-fabricated via brown alga *F. vesiculosus* against Gram (+) bacteria. R1: *Streptococcus mutans* ATCC 25175; R2: *Lactobacillus acidophilus* CH-2, R3: *Staphylococcus aureus* ATCC6538; R4: *Staphylococcus epidermidis*; R5: *Staphylococcus aureus*. Bars: represent stander error. The different letters represent a significant value of means.

**Figure 10:**
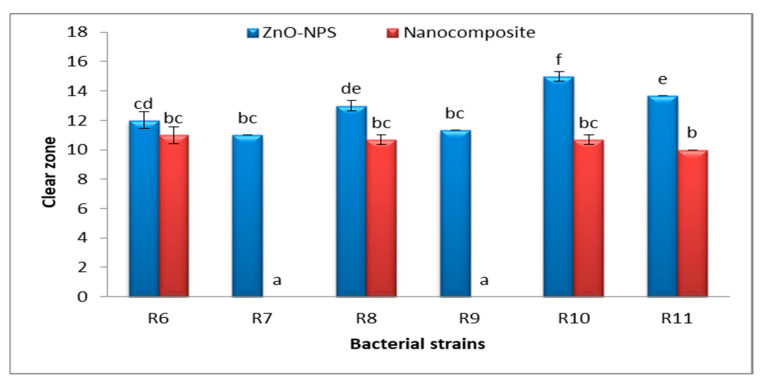
Antibacterial activity of Fu/ZnO-NPs and Fu/ZnO/Alg-NCMs bio-fabricated via brown alga *F. vesiculosus* against Gram (-) bacteria. Bars: represent stander error. The different letters represent significant values of means. R6: *Klebsiella pneumoniae* KY856924; R7: *Acinetobacter* KY856930; R8: E coli KY856932; R9: *Enterobacter aerogenes*; R10: *E coli* KY856933; R11: *Enterobacter* KY856934.

**Figure 11:**
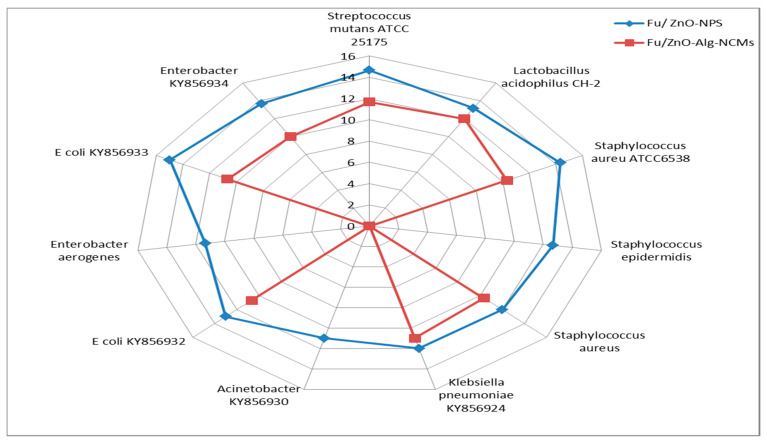
Radar analysis shows the inhibition zone (mm) of both Fu/ZnO-NPs and Fu/ZnO-Alg-NCMs bio-fabricated via brown alga *F. vesiculosus* against MDR bacterial strains.

The antibacterial activity of Fu/ZnO-NPs and Fu/ZnO-Alg-NCMs bio-synthesized via brown alga *F. vesiculosus* towards Gram-positive bacteria (*Lactobacillus acidophilus* CH-2, *Streptococcus mutans* ATCC 25175, *Staphylococcus aureus* ATCC6538, *Staphylococcus aureus, and Staphylococcus epidermidis*), and Gram-negative bacteria (*Klebsiella pneumoniae* KY856924, *Acinetobacter* KY856930, *E coli* KY856932, *Enterobacter aerogenes*, *E coli* KY856933 and *Enterobacter* KY856934) was evaluated by the disc diffusion agar approach (Figure 9, Figure 10 and Figure 11). Fu/ZnO-NPs were found to have greater antibacterial activity than Fu/ZnO-Alg-NCMs against Gram-negative and Gram-positive bacteria, and there was a significant difference in the effects of both Fu/ZnO-NPs and Fu/ZnO/Alg-NCMs against all tested bacterial strains. Results show that Fu/ZnO/Alg-NCMs had no effect on *Staphylococcus epidermidis*, *Acinetobacter* KY856930, and *Enterobacter aerogenes*, whereas there was an apparent effect of ZnO-NPs against the same strains.

**Figure 12:**
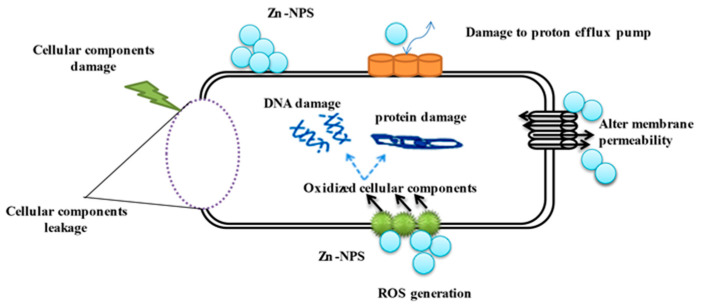
Probable mechanisms of the Fu-ZnO-NPs against bacteria.

Results demonstrate the Fu/ZnO-NPs have more antibacterial activities than Fu/ZnO/Alg-NCMs. This may be due to alginate polymers that coated zinc oxide nanoparticles and reduced the toxicity of Fu/ZnO-NPs due to poor solubility. By coating nanoparticles with an appropriate polymer, the cytotoxicity of nanoparticles can be reduced since they are less harmful due to their poor solubility and prolonged release [74]. Bakil et al. [75] reported the ZnO incorporated with sodium alginate demonstrated slightly stronger antibacterial effects on *Staphylococcus aureus* than on *E. coli*. As a result, sodium alginate (SA)-Zinc oxide (ZnO) nanoparticle has the potential to be used in biomedical applications as a material for wound healing. Bio-fabricated ZnO-NPs may be efficient versus *Staphylococcus aureus* and *Escherichia coli*. Moreover, those bio-synthesized by *Sargassum wightii* inhibited the pathogenic bacteria’s growth, including *Escherichia coli*, *Bacillus subtilis*, *Staphylococcus epidermidis*, *Salmonella typhi*, *Enterococcus faecalis*, *Tyrophyton simii*, *S. aureus*, *Aspergillus niger*, *Cochliobolus lunata*, *Aspergillus flavus*, and *Candida albicans* [76,77]. The alginate/silica/zinc oxide nano-composite effectively inhibits bacteria [76]. The alginate-montmorillonite/lemon essential oil nano-composite was more effective versus Gram-positive bacteria (*Bacillus cereus* and *Bacillus aureus*) than Gram-negative bacteria (*Staphylococcus enteritis* and *Escherichia coli*) [78]. The ZnO–Alginate nano-composite demonstrated the most significant reduction activity versus *Staphylococcus aureus* and *Escherichia coli* [55,57]. In addition, Zinc oxide–sodium alginate–cellulose nano-composite fibers (ZnO–SACNF) showed exceptional antibacterial activity against *E. coli* [79]. Figure 12 demonstrates the possible mechanisms of ZnO-NPs against bacteria due to the distraction of the cell membrane, binding to DNA and proteins, and generation of reactive oxygen species (ROS) [80]. After 15 min of bacterial cell exposure to the ZnO-NPs, 70% of the cytoplasmic membrane was damaged [81]. The mechanisms of ZnO-NPs as antibacterial, including the incorporation of NPs due to loss of proton motive force, internalization of cell walls due to ZnO-localized contact, increased membrane permeability, and ingestion of hazardous dissolved zinc ions, have been greatly influenced by ROS. These have resulted in mitochondrial weakening, intracellular leakage, and oxidative stress gene expression that eventually inhibited cell development and caused cell death [82]. At low doses, ZnO nanoparticles showed deadly effects on *Campylobacter jejuni* and impressive antibacterial activity. Considerable morphological alterations, detectable membrane leakage, and considerable elevations (up to 52-fold) in oxidative stress gene expression were all brought on by ZnO nanoparticles in *Campylobacter jejuni*. ZnO alters the permeability of the membranes through which nanoparticles penetrate and cause oxidative stress in bacterial cells, which ultimately leads to cell death [83].

Fu-ZnO-NPs internalize into the bacterial cell and translocate, penetrating through pores in the cell wall. Reactive oxygen species (ROS) are produced by the redox reaction nanoparticles; ROS oxidizes cellular components, including DNA and protein. The effect on membrane permeability caused cellular component leakage and damage.

## 4. Conclusions

The current work demonstrated brown alga *Fucs. vesiculosus* is a good candidate for the synthesis of Fu/ZnO-NPs and Fu/Alg-ZnO-NCMs, confirmed by FT-IR. The results by XRD, zeta potential, and TEM demonstrate Fu/ZnO-NPs are crystalline, rod-shaped, and have a negative charge. Fu/Alg-ZnO-NCMs are semi-crystalline, spherical, and have more negative charge than Fu/ZnO-NPs. The peak of Zn (EDS analysis) appears in both Fu/ZnO-NPs and Fu/Alg-ZnO-NCMs at 1 KeV, 8.6 KeV, and 9.5 KeV and proves the purity of Fu/ZnO-NPs. The change in shape, charge, and crystalline in Fu/Alg-ZnO-NCMs is due to alginate that acts as a capping agent of Fu/ZnO-NPs. Fu/ZnO-NPs have the best antibacterial activities against MDR bacterial strains (*Streptococcus mutans ATCC 25175; Lactobacillus acidophilus CH-2, Staphylococcus aureus ATCC6538; Staphylococcus epidermidis; Staphylococcus aureus, Klebsiella pneumoniae KY856924: Acinetobacter KY856930; E coli KY856932; Enterobacter aerogenes; E coli KY856933; Enterobacter KY856934),* then that was coated with alginate (Fu/Alg-ZnO-NCMs).

## Figures and Tables

**Table 1:** FT-IR analysis of Fu/ZnO-NPs and Fu/Alg-ZnO-NCMs bio-synthesized via brown alga F. vesiculosus.

Wavenumber cm^−1^	Fu/ZnO-NPS	Fu/Alg-ZnO-NCMs	Assignment	References
3421	+	+31	OH symmetric stretching	[35]
2924	+	−2	OH stretching	[36]
2854	+	+1	C–H stretching	[37]
1655	+	+	amide I-III band	[38]
1634	+	−2	amide I-III band	[38]
1549	+	_	N–H deformation	[39]
1463	_	+	O–H bending	[40]
1439	+	+6	C single bond H deformation	[41]
1405	_	+	CH2 bending vibration	[42]
1266	_	+	Amide III	[43]
1149	_	+	Glycosidic bonds	[44]
1082		−19	C single bond H	[45]
931	_	+	C single bond H out-of-plane	[46]
875	+	−2	C–H stretching vibrations	[47]
843	+	_	–OH groups	[48]
775	_	+	C-H	[49]
710	_	+	N-Cl stretching bond	[50]
606	+	_	ring deformation of phenyl	[51]
582	_	+	M-N stretching vibrations of the complexes.	[52]
565	+	−33	C–C–C deformation of the phenyl ring	[53]
472	_	+	C–C torsion vibrations	[54]
433	+	_	Metal oxygen	[55]

(_) Not detected, (+) detected.

**Table 2:** X-ray diffraction analysis of Fu/ZnO-NPs bio-fabricated via *F. vesiculosus*.

2-Theta °	Area	Cry Size D (nm)	Intensity%	d Value (Angstrom)
31.97	17.09386	26.8	62.4	2.79713
34.579	13.82135	31.5	58.3	2.59188
36.388	28.63312	29.9	100	2.46708
47.745	7.448441	25.3	24.6	1.90339
56.814	11.46893	34.5	46.6	1.61918
63.009	9.757796	33.7	37.2	1.47407
66.33	1.604286	26.7	7.1	1.4081
68.072	10.56759	25.2	29	1.37624
69.174	3.084169	50.6	6.12	1.35699

**Table 3:** XRD analysis of Fu/ZnO/Alg-NCMs bio-fabricated by *F. vesiculosus*.

2-Theta°	Area	Cry Size (D) (nm)	Intensity %	d Value (Angstrom)
23.159	67.99954	0.7	83.8	3.83746
29.457	2.635086	21.7	100	3.0298
31.844	9.625473	1.3	66.2	2.80792
39.564	21.01589	0.7	50.9	2.27601

## Data Availability

All data from this study are presented in the paper.
